# Epidemiological and Clinical Features of Brucellosis in the Country of Georgia

**DOI:** 10.1371/journal.pone.0170376

**Published:** 2017-01-20

**Authors:** Tamar Akhvlediani, Christian T. Bautista, Natalia Garuchava, Lia Sanodze, Nora Kokaia, Lile Malania, Nazibrola Chitadze, Ketevan Sidamonidze, Robert G. Rivard, Matthew J. Hepburn, Mikeljon P. Nikolich, Paata Imnadze, Nino Trapaidze

**Affiliations:** 1 U.S. Army Medical Research Directorate-Georgia (USAMRD-G), Tbilisi, Georgia, United States of America; 2 Walter Reed Army Institute of Research (WRAIR), Silver Spring, Maryland, United States of America; 3 National Center for Disease Control and Public Health, Tbilisi, Georgia, United States of America; 4 Virsaladze Scientific-Research Institute of Medical Parasitology and Tropical Medicine, Tbilisi, Georgia, United States of America; 5 I. Javakhishvili Tbilisi State University, Tbilisi, Georgia, United States of America; 6 U.S. Army Medical Research Institute of Infectious Diseases (USAMRIID), Fort Detrick, Maryland, United States of America; East Carolina University Brody School of Medicine, UNITED STATES

## Abstract

**Background:**

Brucellosis is an endemic disease in the country of Georgia. According to the National Center for Disease Control and Public Health of Georgia (NCDC), the average annual number of brucellosis cases was 161 during 2008–2012. However, the true number of cases is thought to be higher due to underreporting. The aim of this study was to provide current epidemiological and clinical information and evaluate diagnostic methods used for brucellosis in Georgia.

**Methodology:**

Adult patients were eligible for participation if they met the suspected or probable case definition for brucellosis. After consent participants were interviewed using a standardized questionnaire to collect information on socio-demographic characteristics, epidemiology, history of present illness, and clinical manifestation. For the diagnosis of brucellosis, culture and serological tests were used.

**Results:**

A total of 81 participants were enrolled, of which 70 (86%) were from rural areas. Seventy-four percent of participants reported consuming unpasteurized milk products and 62% consuming undercooked meat products before symptom onset. Forty-one participants were positive by the Wright test and 33 (41%) were positive by blood culture. There was perfect agreement between the Huddelston and Wright tests (*k* = 1.0). Compared with blood culture (the diagnostic gold standard), ELISA IgG and total ELISA (IgG + IgM), the Wright test had fair (*k* = 0.12), fair (*k* = 0.24), and moderate (*k* = 0.52) agreement, respectively.

**Conclusions:**

Consumption of unpasteurized milk products and undercooked meat were among the most common risk factors in brucellosis cases. We found poor agreement between ELISA tests and culture results. This report also serves as an initial indication that the suspected case definition for brucellosis surveillance purposes needs revision. Further research is needed to characterize the epidemiology and evaluate the performance of the diagnostic methods for brucellosis in Georgia.

## Introduction

Brucellosis, the most common bacterial zoonosis in both human and animals, has a widespread geographic distribution [1]. Worldwide, approximately 500,000 new human cases of brucellosis are reported annually [2]. Although brucellosis is endemic in many parts of the world, especially in Mediterranean countries, north and east Africa, the Middle East, central Asia and Latin America, this disease often goes unrecognized or unreported.

Brucellosis is caused by small gram-negative coccobacilli of the genus *Brucella*. In 1887, Sir David Bruce, a British military physician was the first to isolate the causative organism from the spleens of patients who died from Mediterranean fever in Malta [3]. The genus *Brucella* consists of seven species, including four that are pathogenic to humans: *B*. *melitensis*, *B*. *abortus*, *B*. *suis*, and *B*. *canis*. In low- and middle-income countries the most common mode of acquiring human brucellosis is through the consumption of contaminated milk or dairy products. Other modes of transmission are through contact and inhalation of organisms from infected animals, principally cattle, goats and sheep [4]. *Brucella* organisms may persist for 5–15 days in milk, 30 days in ice cream, 142 days in butter, and for several weeks in tap water [5–7].

The clinical presentation of brucellosis varies from an acute, nonspecific febrile illness to chronic, debilitating forms whose features may include osteoarticular involvement and neuropsychiatric abnormalities. Although brucellosis can present with signs and symptoms that may raise clinical suspicion, acute brucellosis is often difficult to distinguish from other febrile conditions [4]. Brucellosis diagnosis is mainly based on a history of a possible exposure, microbiological evidence (blood culture and biochemical values), and serological tests. Isolation of the organism is considered the gold standard, but *Brucella* isolates are difficult to grow and require special laboratory safety conditions [6]. Thus, diagnosis is often conducted using serological tests; a titer ≥ 1:160 is commonly considered active brucellosis infection in many developing countries [7].

Brucellosis is an endemic disease in Georgia, a small country situated in the South Caucasus region. According to the National Center for Disease Control and Public Health (NCDC), the average annual number of brucellosis cases was 161 during 2008–2012. However, it is believed that the true number of cases is higher due to underreporting. Serological diagnosis of brucellosis in Georgia has always been based on the Huddelston and Wright agglutination tests; this has not changed over the past three decades [8]. The most recent data on brucellosis in Georgia indicate that the rate of disease among household family members of brucellosis cases is 7% [9].

The aims of this study were to: 1) provide current epidemiological information from individuals infected with brucellosis; 2) examine the performance of bacteriological and serological methods for the diagnosis of brucellosis; and 3) evaluate the clinical manifestations that are used in the suspected case definition of brucellosis according to national surveillance guidelines in Georgia.

## Materials and Methods

### Ethics Statement

The study was performed in accordance with the Declaration of Helsinki and all applicable federal regulations governing the protection of human subjects in research. Participation was voluntary and written informed consent was obtained from all participants before enrollment in the study. The study protocol, written informed consent, study questionnaires, and recruitment materials used in this study were approved by the institutional review boards and scientific ethics committees at the NCDC, IRB00002150 (Tbilisi, Georgia); the US Army Medical Research Institute of Infectious Diseases, IRB00004283 (Fort Detrick, MD, USA; FY07-08), and at the Walter Reed Army Institute of Research, IRB00000794 (Silver Spring, MD, USA; WRAIR #1864.

### Study Site and Participants

Patients were enrolled at the Institute of Parasitology and Tropical Medicine (IPTM) in Tbilisi, the capital of Georgia. Historically, IPTM has been the national reference clinic for brucellosis where patients are diagnosed and treated. For this study, adult patients (18 years of age or older) were eligible for participation if they met the suspected or probable case definition for brucellosis. According to Georgian national surveillance guidelines, a suspected case is defined as a case with fever, intermittent or remittent, lasting more than five days and with at least four of the seven following signs or symptoms: sweats and/or rigors, fatigue and/or malaise, hepatomegaly, polylymphadenopathy, osteoarticular and neuromuscular pains, leucopenia, and multiple organ system involvement. A probable case is defined as a suspected case that also includes an epidemiological link (risk factor) associated with brucellosis. In order to include patients with a suspicion of brucellosis we applied a less stringent criterion for suspected cases. That is, patients with laboratory results suggestive of a case of brucellosis were also enrolled.

Participants were not eligible for study entry if they: 1) had an alternative diagnosis; 2) had been treated for brucellosis within the past 365 days; 3) were less than 18 years old; or 4) had a weight less than 50 kg (110 pounds). Participation was voluntary, and participants were recruited by IPTM investigators. Only eligible participants were enrolled through the informed consent process. At enrollment, a blood sample was collected (10 to 30 mL) for laboratory testing. Then participants were confidentially interviewed using a standardized questionnaire to collect information on socio-demographic characteristics (e.g., age, gender, occupation), epidemiology (e.g., consumption of contaminated products, contact with sick animals, knowledge of brucellosis), history of present illness (e.g., antibiotics, health-seeking behavior), and clinical manifestation (e.g., fever, sweats, fatigue).

### Laboratory Analysis

The diagnosis of brucellosis was performed using culture and serological tests. For bacteriology, aerobic and anaerobic bottles of the BD SEPTI-CHEK^™^ culture system were inoculated with 8–10 mL of fresh blood and observed for bacterial growth for up to 28 days at the laboratory of the NCDC in Tbilisi. Suspected isolates were subjected to microbiological tests and real-time polymerase chain reaction (PCR) (Target 1, Idaho Technology Inc.) to identify *Brucella*. AMOS PCR was used to identify the *Brucella* species [10, 11]. For detecting antibodies against *Brucella*, we used the Huddelston and Wright tests. *Brucella abortus* Antigen (BD) was utilized following the manufacturer’s instructions. The Huddelston test is a rapid slide agglutination test, with results expressed as one through four plus (+) signs. Test results are read in two hours; a result of two or more pluses (++) is considered suggestive of active brucellosis infection. The Wright test is a standard tube agglutination test performed using serial dilutions of serum samples, and its results are read after 24 hours’ incubation at 37°C [12]. In Georgia, a cut-off value of ≥ 1:200 is indicative of brucellosis. Because the Huddelston test may provide false-negative results [13], the definitive brucellosis diagnosis at the IPTM is mainly based on the Wright test. In addition to these standard serological tests, serum samples were tested for anti-*Brucella* IgM and IgG antibodies using a commercial enzyme-linked immunosorbent assay (ELISA) test (IBL International, Hamburg, Germany) and an in-house total (IgG+IgM) ELISA test developed at the U.S. Naval Army Medical Research Unit-3 in Cairo, Egypt [14]. ELISA results did not influence treatment decisions for patients with brucellosis.

### Statistical Analyses

Chi-square or Fisher’s exact test were used to compare differences in categorical variables, and the Student *t*-test or Mann-Whitney U test were used for continuous variables. Study variables of interest in our analysis included age, gender, occupation, complaints, signs or symptoms on hospital admission, risk factors for disease, laboratory results (culture and serologic tests), and disease knowledge. Using culture results as the gold standard, we determined the performance and agreement of the Wright test and two ELISA tests. For that, we calculated the sensitivity, specificity, and Kappa values (κ) along with their 95% confidence intervals (95% CI). The strength of agreement was interpreted as poor (κ values < 0); small (κ values 0.0–0.20); fair (κ values 0.21–0.40); moderate (κ values 0.41–0.60); substantial (κ values 0.61–0.80); and almost perfect (κ values 0.81–1.00). All reported *p-values* were two-sided, and *p-values* < 0.05 were considered statistically significant. All analyses were conducted using Epi Info version 3.5.3 (Centers for Disease Controls and Prevention, Atlanta, USA) and Stata version 12.0 (STATA, College Station, TX, USA).

## Results

### Demographic and Epidemiological Data

A total of 81 participants were enrolled at the IPTM from April 2009 to July 2011 (Table 1). At entry, the mean age was 39.9 (standard deviation = 15.1) years and 45% of participants were 21–40 years old. Seventy-four percent of participants were males and most participants (86%) were from rural areas; 79% were from Kakheti (eastern Georgia) or Kvemo-Kartli (southeast of the capital Tbilisi). Half of the participants had completed secondary education, and 12% had completed higher education. The most commonly reported occupations were farmer (25%) and shepherd (21%); 23% of participants listed themselves as unemployed but indicated that they lived on farms and involved in animal husbandry.

**Table 1:** Epidemiological characteristics of the study participants.

Feature	n (%)
Age (years), mean (SD)	39.9 (15.1)
Gender, men	60 (74)
Regions, Kakheti and Kvemo-Kartli (eastern)	64 (79)
Ethnic Georgians	41 (51)
Education, secondary or higher	53 (65)
Occupation, farmer	20 (25)
Consumed undercooked meat products	50 (62)
Consumed unpasteurized dairy products^a^	60 (74)
Direct contact with sick animals	17 (21)
Direct contact with aborted livestock	13 (16)
Brucellosis knowledge	16 (20)
Vaccinate animals against brucellosis	9 (11)
Referred to medical facilities before IPTM	63 (78)

Note: SD, standard deviation; IPTM, Institute of Parasitology and Tropical Medicine; denominators may vary due to missing data.

^a^ Dairy products included milk, yogurt, sour cream, fresh cheese or sheep cheese.

### Risk Factors and Disease Knowledge

Seventy-four percent of the participants reported having consumed unpasteurized milk products within four months before symptom onset and 62% had consumed undercooked meat products. The majority (80%) of participants reported owning livestock. Cattle (58%), sheep (42%), and goats (22%) were the most frequently reported species. Pigs were reported to a lesser extent (11%). Approximately, 37% of participants had direct contact with sick and aborted livestock within four months prior to disease onset. In this study, the occupational risk for brucellosis was similar among participants: 33 (41%) worked on a farm, 36 (44%) sheared sheep, 41 (51%) skinned animals, 41 (51%) slaughtered cattle, and 40 (49%) assisted cattle and/or small ruminants in delivery. In addition, 10 participants (12%) reported hunting, and 5 (6%) had contact with aborted fetuses or tissues. As to travel outside of the country, this was rarely reported (one participant).

Regarding disease knowledge, only 16 participants (20%) knew how brucellosis is transmitted and few participants (n = 5) answered that they took protective measures against brucellosis, although they were not able to define such measures. Only two participants had been vaccinated against brucellosis. Livestock vaccination against brucellosis was reported by a few participants (n = 9).

### Referral to Medical Facilities

Sixty-three (78%) participants sought care in other medical facilities before coming to the IPTM. Of these, 46, 12, and 5 visited one, two, and three other medical facilities, respectively. At the first facility, 29 participants received a diagnosis, of which brucellosis was suspected in 14 (48%) of them. At the second facility, 7 out of 12 participants had a diagnosis, and for 5 (71%) of them this was suspected brucellosis. At the third facility, 2 out of 5 participants received a diagnosis, and 1 of these was suspected brucellosis. In total, 23 (28%) participants were previously diagnosed with clinical suspicion of brucellosis before coming to the IPTM.

### Clinical and Physical Evaluation

Fever was present in all participants at some point during the infection; but 83% of them presented with fever on admission. The most frequent complaints were sweats (92%), joint pain (91%), aches (90%), malaise (90%), fatigue (87%), and rigors (86%) (Table 2). The signs or symptoms most frequently reported on admission were sweats (88%), malaise (87%), general aches (86%), fatigue (73%), and arthralgia (72%). Loss of appetite, weight loss, and arthritis were also observed in 46%, 44%, and 24% of participants, respectively. Other signs or symptoms also associated with brucellosis, such as abdominal pain, pain in the testicles, and splenomegaly, were reported by less than 10% of the participants. In addition, 15 participants (19%) had some type of complication. This included: 5 participants with epididymo-orchitis, 2 with cystitis, 2 with pyelonephritis, 1 with hepatitis, and 1 with acute ileitis.

**Table 2:** Complaints, signs/symptoms, and physical examination of the study participants.

Feature	n (%)		n (%)
Complaints		Physical Examination	
Sweats	73 (92)	Arthritis	18 (22)
Joint pain	72 (91)	Myositis	0 (0)
Aches	69 (90)	Bursitis	3 (4)
Malaise	71 (90)	Neuritis	3 (4)
Fatigue	69 (87)	Pneumonia	0 (0)
Rigors	68 (86)	Hepatomegaly	9 (11)
		Splenomegaly	6 (7)
		Jaundice	1 (1)
Signs or Symptoms			
Sweats	67 (88)	Lymphadenopathy	0 (0)
Malaise	66 (87)	Pyelonephritis	2 (2)
Aches	65 (86)	Hepatitis	1 (1)
Fatigue	55 (73)	Meningitis	0 (0)
Arthralgia	54 (72)	Epididymo-orchitis	5 (6)
Rigors	53(70)	Prostatitis	0 (0)
Loss of appetite	35 (46)	Cystitis	2 (2)
Weight loss	32 (44)	Endocarditis	0 (0)
Myalgia	22 (29)	Multiple organ system involvement*	33 (41%)
Arthritis	18 (24)		
Depression	14 (19)		
Sleep disturbances	13 (17)		
Difficulty concentrating	11 (15)		
Constipation	8 (11)		

Denominators may vary due to missing data.

* Multiple organ system involvement—involvement of more than two organ systems.

### Evaluation of Brucellosis Case Definition

We specifically evaluated clinical manifestations that are used in the suspected case definition of brucellosis according to national guidelines: sweats and/or rigors, fatigue and/or malaise, hepatomegaly, polylymphadenopathy, osteoarticular, and neuromuscular pains, leucopenia, and multiple organ system involvement. As indicated above, sweats and/or rigors and fatigue and/or malaise were commonly reported by participants. At the IPTM, a complete blood count was not routinely conducted for patients treated on an outpatient basis, and thus, leucopenia could not be assessed for case definition. Moreover, during physical examination, hepatomegaly was present in only 9 participants (11%), but it was manually assessed because ultrasound was not available at the IPTM. Results on liver function tests were not collected because these tests are not performed as part of the routine laboratory assessment for suspected brucellosis cases. None of the patients had poly-lymphadenopathy. Assuming that more than two organ systems were involved, 33 (41%) of the participants had multiple organ system involvement (Table 2). According to our analyses, 33 of the 79 (41%) participants initially met the suspected case definition; 2 participants were excluded because of missing data. All suspected cases had an epidemiological exposure suggestive of brucellosis; thus, they were classified as probable cases. Of these, 20 (61%) were positive by the Wright test and 16 (48%) were positive by blood culture. Of the participants that did not meet the suspected case definition (n = 46), 20 and 16 of them were positive by the Wright test and by blood culture, respectively.

### Hospitalization and Antibiotic Use

In our study population, only 12 (15%) participants required hospitalization. The other participants were treated on an outpatient basis at IPTM. Among hospitalized participants, the average time spent in hospital was 18 days (range = 8–27 days). Twenty-five (31%) participants were treated with antibiotics before coming to the IPTM. At the IPTM, doxycycline and rifampin were used for the treatment of uncomplicated brucellosis. Triple therapy with additional streptomycin was used in more severe cases. To our knowledge, no relapses have occurred in our study population.

### Laboratory Results

Among 81 participants, 41 (63%) were positive by the Wright test, and 33(41%) were positive by blood culture. Compared to the blood culture (gold standard), total ELISA (96.88%) and IgG ELISA (94.29%) had the highest sensitivity values (Table 3). In contrast, IgM ELISA (84.78%) and the Wright test (71.74%) had the highest specificity values. Analysis also revealed a perfect agreement between the Huddelston and Wright tests (*k* = 1.0, data not shown). However, IgG ELISA, total ELISA (IgG + IgM), and the Wright test, had fair (*k* = 0.12), fair (*k* = 0.24), and moderate agreement (*k* = 0.52), respectively, when compared to blood culture. Additionally, we found slight agreement between blood culture and the IgM ELISA test (*k* = 0.19) and substantial agreement between total ELISA and IgG ELISA test results (*k* = 0.65).

**Table 3:** Sensitivity, specificity, and Kappa values of serological tests compared with blood culture (the gold standard) for brucellosis diagnosis.

Test	Sensitivity % (95% CI)	Specificity % (95% CI)	Kappa *ƙ* (95% CI)
Wright (a titer ≥ 1:200)	81.82 (65.61–91.39)	71.74 (57.42–82.68)	0.52 (0.30–0.74)
IgM ELISA	33.33 (19.75–50.39)	84.78 (71.78–92.43)	0.19 (-0.001, 0.39)
Total ELISA (IgG+IgM)	96.88 (84.26–99.45)	30.23 (18.60–45.11)	0.24 (0.08, 0.40)
IgG ELISA	94.29 (80.84–99.30)	56.63% (45.29–67.47)	0.12 (-0.01 to 0.25)

Note: ELISA, enzyme-linked immunosorbent assay; CI, confidence interval.

Among blood culture positive cases (n = 33), 32 were positive for *B*. *melitensis*, and 1 for *B*. *abortus*. Interestingly, we observed that 6 (18%) cases who were positive by blood culture were negative by the Wright test. Of these, two cases were scored two pluses by the Huddelston test while the other four cases were negative by the Huddelston test. Besides, in this group of blood culture positive cases (n = 6), one case was negative by total ELISA, and all cases were negative by IgM ELISA. Table 4 shows the correlation between culture and serological tests of these six cases.

**Table 4:** Six patients positive by blood culture, but negative by Wright and/or ELISA tests.

ID	Culture Isolated	Wright Test	Huddelston Test	Total ELISA(IgG+IgM)	IgM	IgG
13	*B*. *melitensis*	Negative	Negative	Negative	Negative	Negative
18	*B*. *melitensis*	1/100	++	≥ 320	Negative	2.27
19	*B*. *melitensis*	Negative	+	≥ 320	Negative	1.37
26	*B*. *melitensis*	Negative	Negative	≥ 640	Negative	3.13
53	*B*. *melitensis*	Negative	Negative	≥ 320	Negative	1.56
68	*B*. *melitensis*	1/100	++	≥ 2560	Negative	1.84

Note: ELISA, enzyme-linked immunosorbent assay.

## Discussion

Since the first documented human case of brucellosis in Georgia was reported by Dr. Makhviladze in 1923 [13], the disease has been detected in several regions of the country. In the following decades, this zoonotic infection became a serious problem for livestock and a constant threat to human health. To our knowledge, few published reports have studied the epidemiology and clinical features of this endemic disease in Georgia [8–9, 15–17].

The findings of the present study clearly indicate that the burden of brucellosis affects mostly males and young adults (21–40 years old), and that infection is more prevalent in Kakheti (a region in eastern Georgia) and Kvemo-Kartli (a region southeast of the capital Tbilisi). In the South Caucasus region, males are more involved in the care and management of farm and domestic animals, and are more likely to be shepherds; this may explain the high disparity in brucellosis prevalence compared to females. A similar finding was also noted in Azerbaijan, a neighboring country [18]. Brucellosis can infect persons of any age, but in endemic areas most cases occur in young adults. This fact is also observed in our study population and is directly associated with occupational exposure. In Georgia, young adults from rural areas often have direct contact with infected or aborted animals, and therefore, have more risk for *Brucella* exposure. The age distribution of brucellosis cases in our population is similar to that described in a previous brucellosis chart review report [8]. Data from that study also indicated that most brucellosis cases were from eastern Georgia, mainly from the regions of Kakheti and Kvemo-Kartli. This is consistent with our findings as three-fourths of the study participants came from those regions.

Transmission of brucellosis to humans is highly dependent upon the *Brucella* species. Among farmers or veterinarians, the common route of *B*. *abortus* transmission is through the placenta, fetus, fetal fluids and vaginal discharges of infected animals. Although *B*. *abortus* can be also transmitted via unpasteurized cow’s milk, it is less pathogenic compared to *B*. *melitensis*, which is predominantly transmitted via consumption of unpasteurized milk or dairy products from sheep and goats [19]. In this study, we found that a high percentage of participants reported having consumed unpasteurized milk products and undercooked meat products. Therefore, it is plausible to suggest that the gastrointestinal route could be of the main mode of brucellosis transmission.

Interestingly, a recent case-control study reporting several risk factors associated with brucellosis such as living in eastern Georgia, sheep ownership and animal-related work, consumption of unpasteurized dairy products was not identified as a risk factor for transmission [16]. In epidemiologic analysis, modeling and variable selection are especially important when several risk factors for disease are considered in a regression model [20]. However, variables or risk factors that are moderately or highly correlated with each other, also described as the problem of collinearity, may result in the omission of a relevant risk factor, yielding an elevated risk of false-negative or spurious results. According to this case-control study, consumption of raw dairy products was a protective risk factor for brucellosis (odds ratio = 0.16; *p*-value = 0.01), even though it occurred significantly more often in cases compared to controls, and is commonly reported in the medical literature [2, 4, 7]. Further comprehensive epidemiological studies with a larger sample size are required to better identify risk factors associated with brucellosis in Georgia.

In human brucellosis, the symptoms and clinical findings depend on the immune response, duration of disease, and age of the patient [21]. Among the brucellosis cases in this study, the most common systemic symptoms were intermittent fever, malaise, joint and back pain, aches, headache, weight loss, and sweats and chills during the evening. The frequency of these signs or symptoms is consistent with that described in the medical literature. Interestingly, 15–19% of the participants reported neuropsychiatric symptoms such as depression, sleep disturbances, and difficulty concentrating that have only rarely been documented among brucellosis cases in the past in Georgia [15]. The true rate of neuropsychiatric manifestations of brucellosis is unknown in Georgia, because most patients do not undergo a full evaluation for the presence of these symptoms, which may also simply be unreported. Despite the rare involvement of the central nervous system by *Brucella* species, Shehata et al. recommend testing for neurological and psychiatric symptoms in brucellosis patients from endemic areas [22].

Bacteriology is accepted as the gold standard for brucellosis diagnosis. However, for many reasons (e.g., time-consuming, insensitive, previous antibiotic treatment), *Brucellae* often cannot be cultured from patients’ samples. Therefore, other tests are used to diagnose brucellosis. Because bacteriological culture for *Brucella* was not performed in Georgia for decades [8], there was only inferred evidence of the predominance of *B*. *melitensis* in the eastern Georgia and *B*. *abortus* in the western part of the country. This inference was primarily based on the livestock production data, in which Kakheti and Kvemo-Kartli are top sheep-producing regions in eastern Georgia, while beef cattle and dairy cattle productions are more common in the western territories. This study is one of the first to use blood culture with the aim of identifying the specific *Brucella* species circulating in the country. According to our culture results, nearly all brucellosis cases were caused by *B*. *melitensis*. Considering that 4 out of 5 participants enrolled in this study were from eastern Georgia, our results support the evidence that *B*. *melitensis* is the most common *Brucella* species causing human brucellosis in this part of the country.

Recently, some authors have recommended a combination of several tests for brucellosis diagnosis in suspected patients. Some suggest the use of blood cultures and/or real-time PCR for confirmation of Wright-positive cases [23], whereas others suggest that if the agglutination test is negative, culture and/or ELISA, and/or real-time PCR should be performed [24]. In developing countries such as Georgia with limited laboratory capabilities for culture and automated culture systems, as well as PCR other tests such as ELISA, offer an alternative for diagnosis [25]. Based on culture results, upon admission six patients with a strong clinical indication of brucellosis infection were positive but negative for the Wright test. This discrepancy may be attributable to insufficient antibody production in the early days of infection in this group of patients. Considering time, cost and cultivation problems, we recommend that, in suspected brucellosis cases, agglutination tests should be considered the first choice; culture and/or ELISA should be used for those cases with negative agglutination results. This recommendation is in agreement with recently published studies [24, 26].

Based on laboratory results, the Wright test had better agreement compared to both IgM ELISA and total ELISA (IgG + IgM) when culture was used as the gold- standard lab test for brucellosis diagnosis. However, negative culture does not always exclude the diagnosis of brucellosis. Interestingly, we found that total ELISA detected culture positive cases that were negative by the Wright test. This demonstrates the advantage of total ELISA in such cases. In addition, we found that IgM ELISA had poor agreement with blood culture in the diagnosis of brucellosis.

Our analyses also revealed that the suspected case definition for brucellosis was not sensitive enough to capture acute cases, since only 41% of the participants met the suspected case definition. Consequently, we recommend revision of the suspected brucellosis case definition that is currently used for surveillance purposes in Georgia. In particular, the list of signs or symptoms currently used to determine the presence of a suspected case should be modified because many of these signs and symptoms are rarely reported (e.g., poly-lymphadenopathy) or are simply difficult to evaluate (e.g., leucopenia) due to limitations of laboratory capacity in Georgia. A robust case definition for brucellosis is important for surveillance, clinical research, and outbreak investigations.

This study had some limitations. First, most participants were from eastern Georgia, thus our findings cannot be generalized to represent the burden of brucellosis in other regions of the country. Second, although this study was conducted at the IPTM, the national reference clinic for brucellosis in Georgia, we suspect that most patients with acute infection were treated at their local medical facilities where findings on risk factors, brucellosis knowledge, animal vaccination status, and so on might differ from ours. Finally, information on signs or symptoms may be biased because of recall bias among participants. Despite these limitations, the present study provides additional information on the epidemiological and clinical characteristics of this endemic disease in Georgia. Further, this was first study to evaluate the performance of two ELISA tests (commercial and in-house) for brucellosis diagnosis in the country.

## Conclusion

In summary, we found that brucellosis infection was more likely to occur in males, young adults aged 21–40 years, and from individuals from eastern Georgia. The high percentage of participants that reported having consumed unpasteurized milk products and undercooked meat products would suggest that the gastrointestinal route is the main mode of brucellosis transmission. In addition, *B*. *melitensis* was the most common *Brucella* species found in the study population, and the Wright test had a better agreement with blood culture results than did two ELISA (in house and commercial) tests. Further research is needed to characterize the epidemiology of brucellosis and to elucidate the probable routes of transmission with the aim of understanding the etiology of brucellosis and informing prevention efforts in Georgia. Our study also provides initial evidence that the suspected case definition for brucellosis surveillance purposes needs to be revised.

## Supporting Information

S1 Table Tables for data analysis. (XLSX)
